# Circulating uric acid levels and subsequent development of cancer in 493,281 individuals: findings from the AMORIS Study

**DOI:** 10.18632/oncotarget.16198

**Published:** 2017-03-15

**Authors:** Andrew Yiu, Mieke Van Hemelrijck, Hans Garmo, Lars Holmberg, Håkan Malmström, Mats Lambe, Niklas Hammar, Göran Walldius, Ingmar Jungner, Wahyu Wulaningsih

**Affiliations:** ^1^ King's College London, Division of Cancer Studies, Cancer Epidemiology Group, London, UK; ^2^ Regional Cancer Centre, Uppsala, Sweden; ^3^ Department of Surgical Sciences, Uppsala University Hospital, Uppsala, Sweden; ^4^ Unit of Epidemiology, Institute of Environmental Medicine, Karolinska Institutet, Stockholm, Sweden; ^5^ Department of Medical Epidemiology and Biostatistics, Karolinska Institutet, Stockholm, Sweden; ^6^ AstraZeneca R&D, Mölndal, Sweden; ^7^ Unit of Cardiovascular Epidemiology, Institute of Environmental Medicine, Karolinska Institutet, Stockholm, Sweden; ^8^ Department of Medicine, Clinical Epidemiological Unit, Karolinska Institutet and CALAB Research, Stockholm, Sweden

**Keywords:** uric acid, cancer, prospective study

## Abstract

**Objectives:**

Serum uric acid has been suggested to be associated with cancer risk. We aimed to study the association between serum uric acid and cancer incidence in a large Swedish cohort.

**Results:**

A positive association was found between uric acid levels and overall cancer risk, and results were similar with adjustment for glucose, triglycerides and BMI. Hazard ratio (HR) for overall cancer for the 4^th^ quartile of uric acid compared to the 1^st^ was 1.08 (95% CI: 1.05–1.11) in men and 1.12 (1.09 – 1.16) in women. Site-specific analysis showed a positive association between uric acid and risk of colorectal, hepatobiliary, kidney, non-melanoma skin, and other cancers in men and of head and neck and other cancers in women. An inverse association was observed for pulmonary and central nervous system (CNS) cancers in men and breast, lymphatic and haematological, and CNS malignancies in women.

**Materials and Methods:**

We included 493,281 persons aged 20 years and older who had a measurement of serum uric acid and were cancer-free at baseline in the AMORIS study. Multivariable Cox proportional hazards regression was used to investigate sex-specific quartiles of serum uric acid in relation to cancer risk in men and women. Analysis was further adjusted for serum glucose, triglycerides and, where available, BMI. Site-specific analysis was performed for major cancers.

**Conclusions:**

Altered uric acid levels were associated with risk of overall and some specific cancers, further indicating the potential role of uric acid metabolism in carcinogenesis.

## INTRODUCTION

Uric acid is the product of the metabolism of dietary or endogenous adenine-based and guanine-based purines, and is excreted by the kidney and gut [1, 2]. Uric acid has paradoxically been found to have the characteristic of being an antioxidant in the extracellular environment, whilst having prooxidative effects in the intracellular environment [3, 4]. As an antioxidant, uric acid acts as a scavenger of oxygen radicals, and thus may serve to reduce carcinogenic reactive oxygen species (ROS) [2, 5]. ROS are carcinogenic as they increase the mutation rate in cells, and therefore increase their oncogenic potential [5, 6]. As a prooxidant, uric acid contributes to tumourigenesis by entering normal cells and promoting tumour cell proliferation, migration, and survival, mediated by ROS and inflammatory stress [7]. Recent epidemiological studies looking at the association of serum uric acid and cancer incidence have found conflicting results. A cohort study of 354,110 participants in Taiwan supported the hypothesis that uric acid was cancer-protective as it suggested that low serum uric acid levels were associated with a higher risk of all cancer mortality relative to high serum uric acid levels [8]. Conversely, a cohort study of 83,683 participants in Austria found that high levels of serum uric acid were linked to a higher risk of all cancer mortality [9]. Furthermore, a cohort study of 8,274 participants in China found that incidence of cancer increased with serum uric acid levels, but only in females with diabetes [10]. A recent systematic review and meta-analysis of 17 studies on the link between serum uric acid and cancer incidence and mortality was inconclusive [2].

A link between uric acid and cancer that involves metabolic syndrome (MetS) has also been speculated. MetS is characterised by insulin resistance, hypertension, abnormal lipids, and chronic inflammation [11]. MetS is associated with higher frequencies of elevated serum uric acid levels (hyperuricemia), and, likewise, hyperuricemia is associated with subsequent MetS [12, 13]. Furthermore, studies using genetic uric acid scores as instrumental variables suggested uric acid has a bystander role in MetS [14]. Despite this strong relationship, the effect of MetS on the association between uric acid and cancer has not yet been explored.

High consumption of animal protein and seafood have been associated with higher prevalence of hyperuricemia [15]. Such consumption habits may reflect an unhealthy diet and lifestyle [16], suggesting serum uric acid is an inverse marker of health. Several studies have shown that high serum uric acid is an independent risk factor for hypertension [17], diabetes [18], CVD, and mortality [19].

We studied the association between serum uric acid levels and the incidence of overall and site-specific cancer in the Swedish Apolipoprotein-related MOrtality RISk study (AMORIS) including 493,281 adults, who were followed-up for up to 25 years. Additionally, we took into account serum glucose and triglycerides levels to account for the potential confounding by MetS on the association between circulating uric acid and cancer risk.

## RESULTS

A total of 72,349 persons developed cancer during follow up (mean: 19.47 years). More cancers were observed in men (56.96%). The mean age of participants at baseline was 45 years. The majority of the study population (90.7%) was gainfully employed (Table 1). Overall, participants with cancer diagnosis during follow-up had higher baseline uric acid levels compared to the group without cancer.

**Table 1:** Descriptive characteristics of study population by cancer status

	No Cancer N=420,932	Cancer N=72,349
**Age (years)**		
Mean (SD)	43.22 (13.75)	52.56 (11.51)
**Gender** – N (%)		
Male	223592 (53.12)	41207 (56.96)
Female	197340 (46.88)	31142 (43.04)
**Education Level** – N (%)		
Low	102391 (25.43)	22088 (32.02)
Medium	182446 (45.31)	28896 (41.90)
High	117833 (29.26)	17988 (26.08)
**Socioeconomic Status** – N (%)		
White collar	194672 (46.25)	37281 (51.53)
Blue collar	185694 (44.11)	29787 (41.17)
Not gainfully employed/missing	40566 (9.64)	5281 (7.30)
Serum Uric Acid* – N (%)		
Quartile 1	105080 (24.96)	15770 (21.80)
Quartile 2	107181 (25.46)	17114 (23.65)
Quartile 3	105913 (25.16)	18528 (25.61)
Quartile 4	102758 (24.41)	20937 (28.94)
**Follow up time (years)**		
Mean (SD)	20.51 (5.35)	13.43 (6.68)
**CCI category** – N (%)		
0	399736 (94.96)	67444 (93.22)
1	15218 (3.62)	3597 (4.97)
2	3666 (0.87)	795 (1.10)
3+	2312 (0.55)	513 (0.71)

* Serum uric acid quartiles (μmol/L): Quartile 1 (<281), quartile 2 (281-319), quartile 3 (319-362), quartile 4 (>362) in men, quartile 1 (<207), quartile 2 (207-240), quartile 3 (240-279), quartile 4 (>279) in women. CCI = Charlson comorbidity. index.

Higher cancer risk was observed with increasing levels of serum uric acid (Table 2), with a HR of 1.09 (95%CI: 1.06 – 1.13) for each log unit increase of serum uric acid in the multivariable model. Results were similar with the crude levels of uric acid (data not shown). Further adjustment for serum glucose and TG showed similar results (e.g. HR for overall cancer for each log unit increase of serum uric acid was 1.07 (95%CI: 1.03 – 1.11)). Analysis in the subgroup with information of BMI showed similar albeit weaker results owing to smaller sample size, for instance, HR for every log unit increase of serum uric acid was 1.11 (95%CI: 1.00 – 1.24)) whereas the trend for the quartiles of serum uric acid was weaker (P_trend_ = 0.07). No marked difference was seen with adjustment for BMI. A sensitivity analysis excluding participants with follow up less than 2 years was consistent with these results (Supplementary Table 2, Supplementary Information). A positive association was also found between uric acid levels and risk of death (Supplementary Table 3, Supplementary Information). When analysis was stratified by gender, similar associations were observed in men (e.g. HR for log unit increase of serum uric acid: 1.18 (95%CI: 1.12 – 1.24)) and women (e.g. HR for log unit increase of serum uric acid: 1.23 (95%CI: 1.17 – 1.29)), although we found a statistically significant interaction between serum uric acid level and gender (Table 3).

**Table 2:** Hazard ratios (HR) and 95% Confidence Intervals (95%CI) for the risk of cancer by log and sex-specific quartiles of serum uric acid

	HR (95%CI)			
	Model 1	Model 2*	Model 3^†^	Model 4^§^
N (Cancer/Total Participants)	72,349/493,281	72,349/493,281	72,349/493,281	9,447/66,931
Log serum uric acid	1.94 (1.88 – 2.00)	1.09 (1.06 – 1.13)	1.07 (1.03 – 1.11)	1.11 (1.00 – 1.24)
Serum uric acid				
Quartile 1	1.00 (Ref)	1.00 (Ref)	1.00 (Ref)	1.00 (Ref)
Quartile 2	1.05 (1.03 – 1.07)	1.00 (0.98 – 1.03)	1.00 (0.98 – 1.03)	0.98 (0.92 – 1.03)
Quartile 3	1.16 (1.14 – 1.19)	1.01 (0.99-1.03)	1.01 (0.99 – 1.03)	1.01 (0.96 – 1.08)
Quartile 4	1.46 (1.43 – 1.49)	1.05 (1.03 – 1.07)	1.04 (1.02 – 1.06)	1.05 (0.99 – 1.11)
P_trend_	<0.0001	<0.0001	0.0002	0.07

*Adjusted for age, gender, education level, SES and CCI category.

^†^Adjusted for age, gender, education level, SES, CCI category, serum glucose and triglycerides.

^§^Adjusted for age, gender, education level, SES, CCI category and BMI in the subgroup with BMI.

**Table 3:** Gender stratified hazard ratios (HR) and 95% Confidence Intervals (95%CI) for the risk of cancer by log and sex-specific quartiles of serum uric acid

	No. of cancer/Total participants	Men HR (95%CI)	No. of cancer/Total participants	Women HR (95%CI)
Total group	41207/264799		31142/228482	
Log serum uric acid		1.18 (1.12 – 1.24)		1.23 (1.17-1.29)
Serum uric acid				
Quartile 1	9471/64570	1.00 (Ref)	6299/56280	1.00 (Ref)
Quartile 2	9945/67089	1.00 (0.97 – 1.03)	7169/57206	1.03 (1.00 – 1.07)
Quartile 3	10596/66812	1.05 (1.02 – 1.07)	7932/57629	1.03 (0.99 – 1.06)
Quartile 4	11195/66328	1.08 (1.05 – 1.11)	9742/57367	1.12 (1.09 – 1.16)
P_trend_		<0.0001		<0.0001
P_interaction_				<0.0001

All models were adjusted for age, education level, SES and CCI category.

Tables 4 and 5 show the association between serum uric acid levels and different site-specific cancers in men and women, respectively. In men, statistically significant positive associations were found for colorectal, hepatobillary, kidney, and non-melanoma skin cancers, and statistically significant inverse associations were found for pulmonary cancers. In women, statistically significant positive associations were found for head and neck cancers, and statistically significant inverse associations were found for breast, and lymphatic and haematological cancers in women. The hazard ratios (HRs) for positive associations were the largest for hepatobillary cancers in men (e.g. HR for log unit increase of serum uric acid: 2.03 (95%CI: 1.38 – 2.99)) (Table 4) and for head and neck cancers in women (e.g. HR for log unit increase of serum uric acid: 1.90 (95%CI: 1.24 – 2.92)) (Table 5). The inverse HRs were the largest for CNS cancers in both men and women (e.g. HR for log unit increase of serum uric acid: 0.60 (95%CI: 0.43 – 0.83) and 0.67 (95%CI: 0.50 – 0.89), respectively).

**Table 4:** Hazard ratios (HR) and 95% Confidence Intervals (95%CI) for the risk of different types of cancer by log and categories of serum uric acid in men

Site-specific cancer	Log Serum Uric Acid	Quartile 1	Quartile 2	Quartile 3	Quartile 4	P_trend_
Prostate						
Events – n		3673	3860	4058	3988	
HR (95%CI)	0.92 (0.85 – 1.00)	1.00 (Ref)	0.97 (0.93 – 1.02)	1.00 (0.96 – 1.05)	1.02 (0.97 – 1.06)	0.08
Pulmonary						
Events – n		745	714	746	756	
HR (95%CI)	0.79 (0.66 – 0.95)	1.00 (Ref)	0.89 (0.80 – 0.99)	0.91 (0.82 – 1.00)	0.93 (0.84 – 1.03)	0.03
Colorectal						
Events – n		897	1012	1162	1269	
HR (95%CI)	1.55 (1.33 – 1.81)	1.00 (Ref)	1.05 (0.96 – 1.15)	1.17 (1.07 – 1.28)	1.31 (1.20 – 1.42)	<0.0001
Gastroesophageal						
Events – n		290	319	336	341	
HR (95%CI)	1.04 (0.79 – 1.38)	1.00 (Ref)	1.02 (0.87 – 1.20)	1.05 (0.90 – 1.23)	1.08 (0.92 – 1.27)	0.83
Hepatobillary						
Events – n		143	142	159	241	
HR (95%CI)	2.03 (1.38 – 2.99)	1.00 (Ref)	0.92 (0.73 – 1.16)	1.00 (0.80 – 1.26)	1.56 (1.27 – 1.92)	0.0001
Pancreas						
Events – n		188	219	210	258	
HR (95%CI)	1.38 (0.98 – 1.94)	1.00 (Ref)	1.08 (0.89 – 1.32)	1.01 (0.83 – 1.23)	1.26 (1.05 – 1.52)	0.19
Kidney						
Events – n		220	247	276	303	
HR (95%CI)	1.52 (1.11 – 2.08)	1.00 (Ref)	1.05 (0.87 – 1.25)	1.13 (0.95 – 1.35)	1.27 (1.06 – 1.51)	0.02
Bladder						
Events – n		569	593	647	718	
HR (95%CI)	1.30 (1.06 – 1.59)	1.00 (Ref)	0.97 (0.86 – 1.08)	1.03 (0.92 – 1.15)	1.16 (1.04 – 1.30)	0.10
Head and neck						
Events – n		223	200	214	280	
HR (95%CI)	1.48 (1.06 – 2.08)	1.00 (Ref)	0.83 (0.69 – 1.01)	0.87 (0.72 – 1.05)	1.16 (0.97 – 1.38)	0.08
Melanoma						
Events – n		428	479	505	503	
HR (95%CI)	1.17 (0.93 – 1.48)	1.00 (Ref)	1.04 (0.91 – 1.18)	1.07 (0.94 – 1.21)	1.09 (0.95 – 1.23)	0.19
Non-melanoma Skin						
Events – n		416	476	521	630	
HR (95%CI)	1.59 (1.27 – 1.99)	1.00 (Ref)	1.06 (0.93 – 1.21)	1.14 (1.00 – 1.29)	1.42 (1.25 – 1.60)	0.0007
Central nervous system						
Events – n		260	242	257	213	
HR (95%CI)	0.60 (0.43 – 0.83)	1.00 (Ref)	0.87 (0.73 – 1.03)	0.89 (0.75 – 1.06)	0.74 (0.62 – 0.89)	0.005
Lymphatic and Haematological						
Events – n		658	709	737	782	
HR (95%CI)	1.03 (0.86 – 1.25)	1.00 (Ref)	1.00 (0.90 – 1.11)	1.01 (0.91 – 1.12)	1.09 (0.99 – 1.21)	0.34
Other Cancer						
Events – n		761	733	768	913	
HR (95%CI)	1.23 (1.03 – 1.47)	1.00 (Ref)	0.92 (0.83 – 1.02)	0.99 (0.90 – 1.10)	1.28 (1.16 – 1.41)	0.009

All models were adjusted for age, education level, SES and CCI category.

**Table 5:** Hazard ratios (HR) and 95% Confidence Intervals (95%CI) for the risk of different types of cancer by log and categories of serum uric acid in women

Site-specific cancer	Log Serum Uric Acid	Quartile 1	Quartile 2	Quartile 3	Quartile 4	P_trend_
Breast						
Events – n		2295	2570	2747	2782	
HR (95%CI)	0.94 (0.86 – 1.02)	1.00 (Ref)	1.00 (0.94 – 1.05)	0.99 (0.94 – 1.05)	0.93 (0.88 – 0.98)	0.03
Pulmonary						
Events – n		444	483	519	698	
HR (95%CI)	0.90 (0.74 – 1.08)	1.00 (Ref)	0.97 (0.85 - 1.10)	0.97 (0.85 – 1.10)	1.22 (1.08 – 1.37)	0.35
Colorectal						
Events – n		555	725	784	1077	
HR (95%CI)	0.92 (0.79 – 1.08)	1.00 (Ref)	1.16 (1.04 – 1.30)	1.17 (1.05 – 1.31)	1.50 (1.35 – 1.66)	0.16
Gastroesophageal						
Events – n		91	122	138	215	
HR (95%CI)	1.15 (0.80 – 1.65)	1.00 (Ref)	1.19 (0.91 – 1.56)	1.25 (0.96 – 1.63)	1.79 (1.40 – 2.29)	0.40
Hepatobillary						
Events – n		82	112	118	209	
HR (95%CI)	1.55 (1.06 – 2.27)	1.00 (Ref)	1.23 (0.92 – 1.63)	1.20 (0.91 – 1.60)	1.96 (1.52 – 2.53)	0.17
Pancreas						
Events – n		119	136	171	283	
HR (95%CI)	1.51 (1.09 – 2.10)	1.00 (Ref)	1.02 (0.79 – 1.30)	1.19 (0.94 – 1.50)	1.82 (1.47 – 2.26)	0.08
Kidney						
Events – n		88	104	121	193	
HR (95%CI)	1.83 (1.24 – 2.71)	1.00 (Ref)	1.05 (0.79 – 1.40)	1.14 (0.87 – 1.50)	1.68 (1.31 – 2.16)	0.08
Bladder						
Events – n		118	149	176	265	
HR (95%CI)	1.09 (0.79 – 1.52)	1.00 (Ref)	1.13 (0.88 – 1.43)	1.24 (0.98 – 1.57)	1.74 (1.40 – 2.16)	0.42
Gynaecological						
Events – n		833	890	1016	1337	
HR (95%CI)	1.22 (1.06 – 1.40)	1.00 (Ref)	0.95 (0.86 – 1.04)	1.01 (0.92 – 1.11)	1.23 (1.12 – 1.34)	0.12
Head and neck						
Events – n		75	86	105	160	
HR (95%CI)	1.90 (1.24 – 2.92)	1.00 (Ref)	1.02 (0.75 – 1.39)	1.16 (0.87 – 1.57)	1.65 (1.25 – 2.17)	0.004
Melanoma						
Events – n		314	320	371	337	
HR (95%CI)	0.90 (0.71 – 1.15)	1.00 (Ref)	0.91 (0.78 – 1.06)	0.98 (0.85 – 1.14)	0.83 (0.71 – 0.96)	0.63
Non-melanoma Skin						
Events – n		216	281	324	456	
HR (95%CI)	1.04 (0.81 – 1.33)	1.00 (Ref)	1.16 (0.97 – 1.39)	1.25 (1.05 – 1.49)	1.67 (1.42 – 1.96)	0.20
Central nervous system						
Events – n		228	238	224	213	
HR (95%CI)	0.67 (0.50 – 0.89)	1.00 (Ref)	0.93 (0.77 – 1.11)	0.81 (0.67 – 0.97)	0.71 (0.59 – 0.85)	0.003
Lymphatic and Haematological						
Events – n		404	414	445	595	
HR (95%CI)	0.80 (0.65 – 0.98)	1.00 (Ref)	0.91 (0.79 – 1.05)	0.91 (0.80 – 1.05)	1.13 (1.00 – 1.28)	0.01
Other Cancer						
Events – n		438	539	673	922	
HR (95%CI)	1.78 (1.50 – 2.11)	1.00 (Ref)	1.20 (1.06 – 1.37)	1.52 (1.35 – 1.72)	2.32 (2.07 – 2.59)	<0.0001

All models were adjusted for age, education level, SES and CCI category.

After Bonferroni correction for multiple testing, we found that the associations in colorectal, hepatobillary, and non-melanoma skin cancers remained significant in men, and associations in CNS and other cancers remained significant in women.

## DISCUSSION

We observed that elevated levels of serum uric acid were associated with an increased cancer incidence in comparison to normal values. Adjustment for glucose and TG did not alter the results, and trends were similar when adjusted for BMI. Cancer site specific and gender stratified analysis showed that positive associations between serum uric acid levels and risk of colorectal, hepatobillary, kidney, and non-melanoma skin cancers in men. For women this was observed for head and neck cancers.

Uric acid has been found to act as both an antioxidant and a prooxidant, both of which may affect carcinogenesis. Uric acid behaves as an antioxidant as it scavenges free radical and chelating transitional metal ions by preventing peroxynitrite-induced protein nitrosylation, lipid and protein peroxidation and inactivating tetrahydrobiopterin [20]. Experiments in which uric acid was administered to healthy volunteers found it to decrease ROS production [21]. Conversely, cells exposed to uric acid generate oxidative stress [22, 23]. Production of ROS is associated with local inflammation, impaired nitric oxide (NO) generation, activation of the renin-angiotensin-aldosterone system, insulin resistance, and fat accumulation [20], and promotes tumour cell proliferation, migration, and survival [7]. Tumourigenesis may occur because ROS increase cellular mutation rate, giving rise to oncogenic potential [5, 6].

We observed that elevated levels of serum uric acid were associated with increased incidence at some cancer sites and decreased incidence at other cancer sites, in comparison to normal values, supporting a paradoxical role for uric acid. Cancer site specific and gender stratified analysis showed positive associations between serum uric acid level and risk of colorectal, hepatobillary, kidney, and non-melanoma skin cancers in men, and head and neck cancers in women. Inverse associations between serum uric acid level and risk of pulmonary cancers in men, and breast, and lymphatic and haematological cancers in women were also shown.

In the current study, the positive associations between serum uric acid levels and cancer incidence support the role of uric acid as a prooxidant for the relevant cancer sites. Hyperuricemia is, therefore, a potential risk factor for colorectal, hepatobillary, kidney, non-melanoma skin, and other cancers in men, and for head and neck and other cancers in women. Conversely, hyperuricemia may be protective for pulmonary and CNS cancers in men, and for breast, lymphatic and haematological, and CNS cancers in women. The differing directions of association may imply variation in cellular responses to oxidative stress, or in the extent of involvement of oxidative stress and antioxidants in specific cancer development. Variation in cellular responses has previously been observed in other cellular stressors, for example, heterogeneous responses to anoxia in neurones [24].

Our results show some consistency with other similar studies. A meta-analysis of five prospective cohort studies [25–28] found that hyperuricemia was associated with an increased incidence of overall cancer (relative risk (RR): 1.03 (95%CI: 1.01 – 1.05, P=0.007)) [2]. Stratification by gender in the meta-analysis showed that hyperuricemia was significantly associated to risk of cancer in men, but not in women (RR: 1.05 (95%CI: 1.02 – 1.08) and 1.01 (95%CI: 0.98 – 1.04), for men and women, respectively), however only one study specifically studied women. Furthermore, the meta-analysis suggested substantial heterogeneity between the studies (I^2^: 44.7 for cancer risk in men and women, 53.8 for cancer risk in men) [2]. Stratification by cancer site found a significant positive association between hyperuricemia and lymphatic and haematological cancers only [2]. This is inconsistent with our finding that hyperuricemia may be protective for lymphatic and haematological cancers in women, possibly due to differences in sample populations and sample sizes. The heterogeneity within our results, and between the previously described studies, may also be caused by confounders, in addition to plausible biological processes.

Serum uric acid concentration reflects the balance between uric acid synthesis and excretion. Increased production of serum uric acid may follow consumption of diets high in purines, acute alcohol consumption, chronic fructose consumption, and severe exercise [20]. Impaired function of the kidney, which primarily excretes uric acid, may also cause hyperuricemia [29]. Lifestyle factors and other chronic diseases potentially have an effect on the association between serum uric acid and cancer, for example, MetS is associated with the aforementioned dietary factors that cause increased production of serum uric acid [11, 20]. Reverse causation between uric acid and MetS has also been indicated given the fact that uric acid causes mitochondrial oxidative stress, stimulating accumulation of fat that is independent of excessive caloric intake [11, 30]. As MetS has been suggested as a risk factor for some cancers [26, 31], this complex association implies that serum uric acid levels may also be a result or a proxy for underlying MetS and/or lifestyle risk factors for cancer. Nevertheless, we obtained similar results when we adjusted our analysis for MetS components, serum glucose and triglycerides.

To our knowledge, this is the largest study investigating serum uric acid and cancer risk in both men and women. We were able to account for MetS components by adjustments for serum glucose and lipids. The current study was strengthened by the large number of individuals with prospective measurements of serum uric acid available in the AMORIS database, all measured at one and the same laboratory. Complete follow up information, detailed information on cancer diagnosis, time of death and emigration was available for each participant through the use of national registers. Exposure and outcome information was obtained independently and assessed in an accurate manner [32]. The AMORIS population was selected following analysis of blood samples from health check-ups in non-hospitalised persons and is representative of the general working population of the greater Stockholm area in terms of SES and ethnicity. Over the course of the study, all-cause mortality was about 14% lower in the AMORIS participants than in the general population of the greater Stockholm area, accounting for age, gender and calendar year [32]. The internal validity of our study is unaffected by the selection of a healthy cohort. There were no repeated measurements for serum uric acid available and no information on tumour severity. We did not have data on other lifestyle factors such as alcohol consumption, smoking status and diet for all study participants. Nevertheless, adjustment for CCI was considered as a proxy for other lifestyle-related disorders and did not alter the results. However, residual confounding may still have occurred which may have resulted in underestimation of the associations observed. Genetic information was unavailable for our study population, and it would be interesting to expand future analysis by combining genetic markers for serum uric acid. Finally, we observed higher risk of mortality with higher uric acid levels. Although we have adjusted our analysis for other co-morbidities, lower risks of some cancers with increasing serum uric acid need to be interpreted carefully due to the remaining possibility of competing risk, which is beyond the scope of this study. Future studies needs to address the above limitations to clarify any causal link between circulating uric acid and specific cancers.

In summary, increased levels of serum uric acid level were associated with risk of overall cancer in this large prospective cohort. The different directions of observed associations may corroborate the paradoxical role of uric acid as both an antioxidant and a prooxidant. Nevertheless, further investigations are needed to assess biological mechanisms that may clarify the link between uric acid metabolism and cancer.

## MATERIALS AND METHODS

### Study population and data collection

Blood samples of men and women from the greater Stockholm area were collected by the Central Automation Laboratory (CALAB) between 1985 and 1996. No other clinical data were included in the CALAB database for all participants [33]. The sample population was representative of the general working population of Stockholm. All participants were either healthy individuals undergoing clinical laboratory testing as part of a general health check-up or outpatients referred for laboratory testing. None of the participants were inpatients at the time their blood samples were taken. The linkage of Swedish national registries, using the Swedish 10-digit personal identity number, to the CALAB database formed the AMORIS study and has been described in detail elsewhere [33–39]. Linkage to registries such as the National Cancer Register and the Hospital Discharge Register provided information on socio-economic status (SES), education level, vital status, cancer diagnosis and emigration. After a recent update, the AMORIS study now includes follow up information until 31^st^ December 2011 for 812,073 individuals [40].

We included 493,281 men and women aged 20 and older with baseline measurements of uric acid without any previous diagnosis of cancer. We excluded those with follow up less than 3 months to reduce reverse causation (i.e. serum uric acid levels can be affected by an undiagnosed cancer). An additional sensitivity analysis, in which the first 2 years of follow up were excluded, was also carried out to address the issue of reverse causation (n = 485,820). Follow up time was defined as the time from baseline measurement until date of cancer diagnosis, death from any cause, emigration or end of study (31^st^ December 2011), whichever occurred first. This study complied with the Declaration of Helsinki and was approved by the Ethics Review Board of the Karolinska Institute.

### Definition of outcomes

The outcomes of this study were overall incident cancer and site-specific cancers. Diagnosis of cancer was obtained from the Swedish National Cancer Register. International Classification of Diseases, 7^th^ revision (ICD-7) codes were used to classify major cancer sites (Supplementary Table 1, Supplementary Information).

### Assessment of exposure and covariates

Uric acid was measured by enzymatic uricase method. Coefficients of variation for uric acid determinations were <2.8% at 164 μmol/L (2.76 mg/dL), 2.3% at 470 μmol/L (7.90 mg/dL) and 1.8% at 624 μmol/L (10.49 mg/dL). Due to gender difference in serum uric acid levels [2, 20, 41, 42], we used sex-specific quartiles to classify levels of uric acid into four quartiles. Uric acid levels in men were grouped into quartile 1 (<281 μmol/L), quartile 2 (281-319 μmol/L), quartile 3 (319-362 μmol/L) and quartile 4 (≥362 μmol/L). Uric acid levels in women were grouped into quartile 1 (<207 μmol/L), quartile 2 (207-240 μmol/L), quartile 3 (240-279 μmol/L), and quartile 4 (≥279 μmol/L). We additionally assess uric acid as a continuous variable by converting it logarithmically to ensure the normal distribution of the variable. Glucose was measured enzymatically with a glucoseoxidase/peroxidase method and Triglycerides (TG) were measured enzymatically with a glycerol-phosphate-oxidase after hydrolysis with lipoprotein lipase. Glucose (mmol/L) and triglycerides (TG; mmol/L) were assayed in 489,613 and 493,281 persons, respectively. Weight and height measurements were assessed in 66,931 participants and body mass index (BMI) was calculated. All methods were fully automated with automatic calibration and performed at one accredited laboratory [35].

SES (white collar, blue collar, unemployed or unknown) was based on the national censuses [43]. Education level was categorised into low (less than high school), medium (high school equivalent), and high (higher education). Charlson comorbidity index (CCI) was calculated to account for other diseases given the association between serum uric acid and metabolic diseases. CCI was based on information from the National Patient Register and consisted of 17 groups of diseases with a specific weight assigned to each disease category, which were summed to obtain an overall score between 0 and 3+ corresponding to comorbidity level [43].

### Statistical analysis

Cox proportional hazards regression was used to estimate hazard ratios (HRs) and their 95% confidence intervals (95% CI) of overall cancer incidence by sex-specific quartiles of uric acid as well as the log transformation of uric acid. The assumption of proportionality of hazard was satisfied upon plotting levels of uric acid on Kaplan-Meier curves. In the multivariate model, we adjusted our analysis for age, gender, education level, SES, and CCI category. To assess whether this association was robust against adverse metabolic states, we performed further analyses adjusting for metabolic markers, i.e. serum glucose and triglycerides, and also adjusting for BMI as a continuous variable in the subgroup with information on BMI available.

We further stratified our analyses by gender to assess the incidence of overall cancer and each major cancer site and assessed the remaining cancers as one group (other cancers). We performed a Bonferroni correction, by dividing the significance threshold, 0.05, by the number of cancer sites assessed, fourteen in men and fifteen in women, to account for multiple testing in our cancer site-specific analyses. We also performed a test for interaction by including the product of uric acid levels and gender in the models. To observe association between serum uric acid and mortality, a similar model assessing the association between uric acid levels and risk of overall death was carried out. All analyses were conducted with Statistical Analysis Systems (SAS) release 9.4 (SAS Institute, Cary, NC).

## SUPPLEMENTARY MATERIALS FIGURES AND TABLES


