# Supplementary material for: Circulating uric acid levels and subsequent development of cancer in 493,281 individuals: findings from the AMORIS Study
Source: Oncotarget. 2017 Mar 15;8(26):42332–42. doi: 10.18632/oncotarget.16198 (PMC5522070; doi:10.18632/oncotarget.16198)
Supplement: Supplementary file 1 [file oncotarget-08-42332-s001.pdf]

# Circulating uric acid levels and subsequent development of cancer in 493,281 individuals: findings from the AMORIS Study

## SUPPLEMENTARY TABLES

**Supplementary Table 1: International classification of diseases (ICD) codes used to classify cancer site**

| Cancer site | ICD-7 |
|---|---|
| All cancer | 140-207 |
| Breast | 170 |
| Prostate | 177 |
| Pulmonary (primary) | 162 |
| Colorectal | 153, 154 |
| Gastroesophageal | 150, 151 |
| Hepatobiliary (primary) | 155 |
| Pancreas | 157 |
| Kidney | 180 |
| Bladder | 181 |
| Gynecological | 171-176 |
| Head and neck | 140-148, 160-161 |
| Melanoma | 190 |
| Non-melanoma Skin | 191 |
| Central nervous system (CNS) | 193 |
| Lymphatic and Haematological | 200-207 |
| Other | 192,194-199 |

**Supplementary Table 2: Hazard ratios (HR) and 95% Confidence Intervals (95%CI) for the risk of cancer by log and sex-specific quartiles of serum uric acid in participants with follow up longer than 2 years**

| | HR (95%CI) | | | |
|---|---|---|---|---|
| | Model 1 | Model 2* | Model 3† | Model 4§ |
| N (Cancer/Total Participants) | 68,677/485,820 | 68,677/485,820 | 68,677/485,820 | 9,036/66,156 |
| Log serum uric acid | 1.95 (1.89 – 2.01) | 1.10 (1.06 – 1.14) | 1.07 (1.03 – 1.11) | 1.13 (1.01 – 1.26) |
| Serum uric acid | | | | |
| Quartile 1 | 1.00 (Ref) | 1.00 (Ref) | 1.00 (Ref) | 1.00 (Ref) |
| Quartile 2 | 1.05 (1.03 – 1.08) | 1.01 (0.99 – 1.03) | 1.01 (0.98 – 1.03) | 0.98 (0.92 – 1.04) |
| Quartile 3 | 1.16 (1.14 – 1.19) | 1.01 (0.99 – 1.04) | 1.01 (0.99 – 1.03) | 1.01 (0.96 – 1.08) |
| Quartile 4 | 1.45 (1.42 – 1.48) | 1.06 (1.03 – 1.08) | 1.04 (1.02 – 1.07) | 1.06 (0.99 – 1.12) |
| $P_{trend}$ | <0.0001 | <0.0001 | 0.0001 | 0.05 |

*Adjusted for age, gender, education level, SES and CCI category.
†Adjusted for age, gender, education level, SES, CCI category, serum glucose and triglycerides.
§Adjusted for age, gender, education level, SES, CCI category and BMI in the subgroup with BMI.

**Supplementary Table 3: Hazard ratios (HR) and 95% Confidence Intervals (95%CI) for the risk of death by log and sex-specific quartiles of serum uric acid**

| | HR (95%CI) | | | |
|---|---|---|---|---|
| | **Model 1** | **Model 2*** | **Model 3†** | **Model 4§** |
| N (Death/Total Participants) | 89,791/493,281 | 89,791/493,281 | 89,791/493,281 | 9,430/66,931 |
| Log serum uric acid | 4.21 (4.10 – 4.33) | 1.63(1.58 – 1.68) | 1.53 (1.49 – 1.58) | 1.70 (1.53 – 1.89) |
| Serum uric acid | | | | |
| Quartile 1 | 1.00 (Ref) | 1.00 (Ref) | 1.00 (Ref) | 1.00 (Ref) |
| Quartile 2 | 1.03 (1.01 – 1.05) | 0.96 (0.94 – 0.98) | 0.98 (0.96 – 1.00) | 0.93 (0.88 – 0.99) |
| Quartile 3 | 1.27 (1.25 – 1.30) | 1.03 (1.01 – 1.05) | 1.04 (1.02 – 1.06) | 1.03 (0.97 – 1.09) |
| Quartile 4 | 2.20 (2.16 – 2.25) | 1.26 (1.23 – 1.28) | 1.23 (1.20 – 1.25) | 1.27 (1.20 – 1.35) |
| $P_{trend}$ | <0.0001 | <0.0001 | <0.0001 | <0.0001 |

*Adjusted for age, gender, education level, SES and CCI category.
†Adjusted for age, gender, education level, SES, CCI category, serum glucose and triglycerides.
§Adjusted for age, gender, education level, SES, CCI category and BMI in the subgroup with BMI.

**Supplementary Table 4: CCI distribution by quartiles of uric acid**

| **CCI Category** | **Serum uric acid** | | | |
|---|---|---|---|---|
| | **Quartile 1** | **Quartile 2** | **Quartile 3** | **Quartile 4** |
| 0 (%) | 115,202 (95.33) | 119,276 (95.96) | 118,661 (95.36) | 114,041 (92.20) |
| 1 (%) | 4,158 (3.44) | 3,753 (3.02) | 4,291 (3.45) | 6,613 (5.35) |
| 2 (%) | 908 (0.75) | 843 (0.68) | 932 (0.75) | 1778 (1.44) |
| 3+ (%) | 582 (0.48) | 423 (0.34) | 557 (0.45) | 1,263 (1.02) |
